# Supplementary material for: Auxin-induced signaling protein nanoclustering contributes to cell polarity formation
Source: Nat Commun. 2020 Aug 6;11:3914. doi: 10.1038/s41467-020-17602-w (PMC7410848; doi:10.1038/s41467-020-17602-w)
Supplement: Supplementary file 1 — Supplementary Information [file 41467_2020_17602_MOESM1_ESM.pdf]

Supplementary Information

Auxin-induced signaling protein nanoclustering contributes to cell polarity formation

Pan et al.

Supplementary Figures

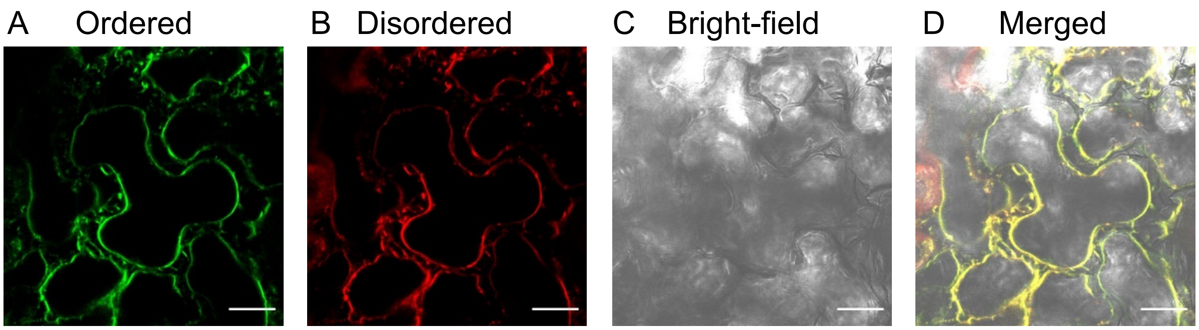

Supplementary Figure 1. Di-4-ANEPPDHQ does not label the cell wall of epidermal pavement cells. Representative images obtained from 2-3-day-old cotyledons stained with di-4-ANEPPDHQ followed by plasmolysis with 0.8 M mannitol for 5 mins. (A) shows di-4-ANEPPDHQ fluorescence recorded between 500-580 nm. (B) shows ANEPPDHQ fluorescence recorded between 620-750 nm. (C) shows the bright-field image. (D) shows the merged image. Scale bars = 15 μm.

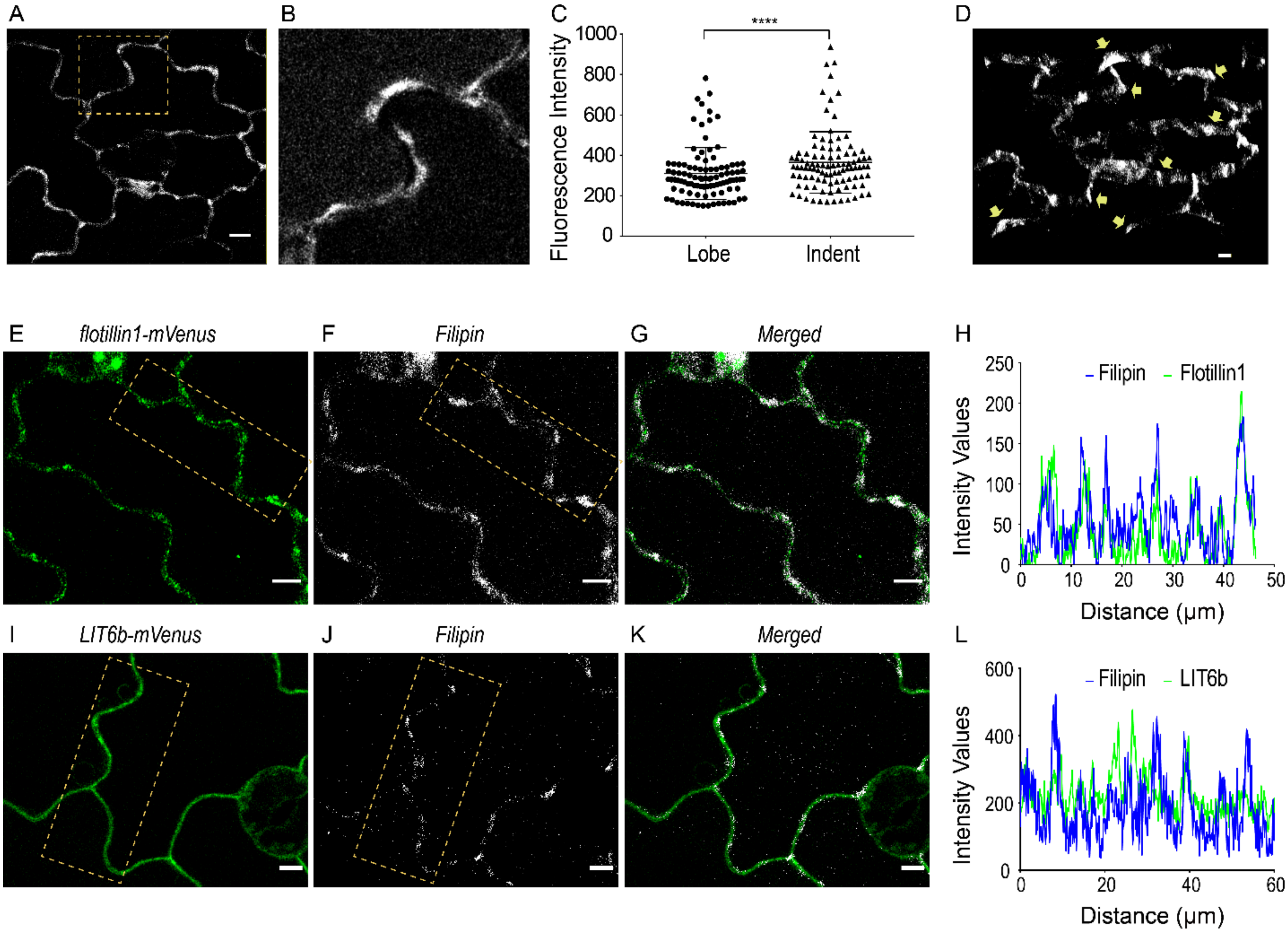

A
B
C
1000
800
600
400
200
0
Fluorescence Intensity
****
Lobe
Indent
D
E
flotillin1-mVenus
F
Filipin
G
Merged
H
250
200
150
100
50
0
Intensity Values
Filipin
Flotillin1
0
10
20
30
40
50
Distance (μm)
I
LIT6b-mVenus
J
Filipin
K
Merged
L
600
400
200
0
Intensity Values
Filipin
LIT6b
0
20
40
60
Distance (μm)

Supplementary Figure 2. Filipin-sterol complexes colocalize with flotillin1 nanoclusters and have an indentation preferred localization. (A-D) Filipin staining of pavement cells (PCs) of 2-3-day-old Arabidopsis cotyledons. (A) Representative confocal images of filipin stained PCs taken at the cell mid-plane in the 2D image. The corresponding 3D reconstructed confocal z stack is shown in (D). The boxed area was further imaged at an optical section slightly above the mid-plane to optically separate complementary lobing and indenting regions on adjacent sides of the cell shown in (B). (C) Quantitative analysis of fluorescence intensity at the complementary lobing and indenting regions of 98 sites of 35 cells from two independent experiments. (D) Oblique view of the z stack 3D reconstruction of filipin stained PCs showing that filipin-sterol complexes appear to be more abundant at lobe-indentation regions (highlighted by yellow arrows) and tricellular junctions. (E-H) Co-detection of filipin-sterol complexes and flotillin1-mVenus particles. (E) Flotillin1-mVenus fluorescence. (F) Filipin-sterol fluorescence. (G) Merged image. Flotillin1-mVenus particles and filipin-sterol complexes showed a very similar punctate pattern on the PM of PCs and therefore appear to be colocalized. (H) The fluorescence intensity along PM in the boxed areas in (E, F) was plotted for flotillin1-mVenus and filipin-sterols. The overlapping fluorescent intensity profiles indicate that flotillin1 nanoclusters indeed colocalize with filipin-sterol complexes. (I-L) Co-detection of filipin-sterol complexes and the PM marker LIT6b-mVenus driven by flotillin1 promoter. (I) LIT6b-mVenus fluorescence. (J) Filipin-sterol fluorescence. (K) Merged image. (L) The fluorescence intensity in the boxed areas in (I, J) was plotted for filipin-sterol and LIT6b-mVenus. In contrast to flotillin1-mVenus and filipin-sterols, the colocalization was not obvious between LIT6b-mVenus and filipin-sterols. Scale bars: 5 μm (A, E, F, G, I, J and K) and 2.5 μm (D).

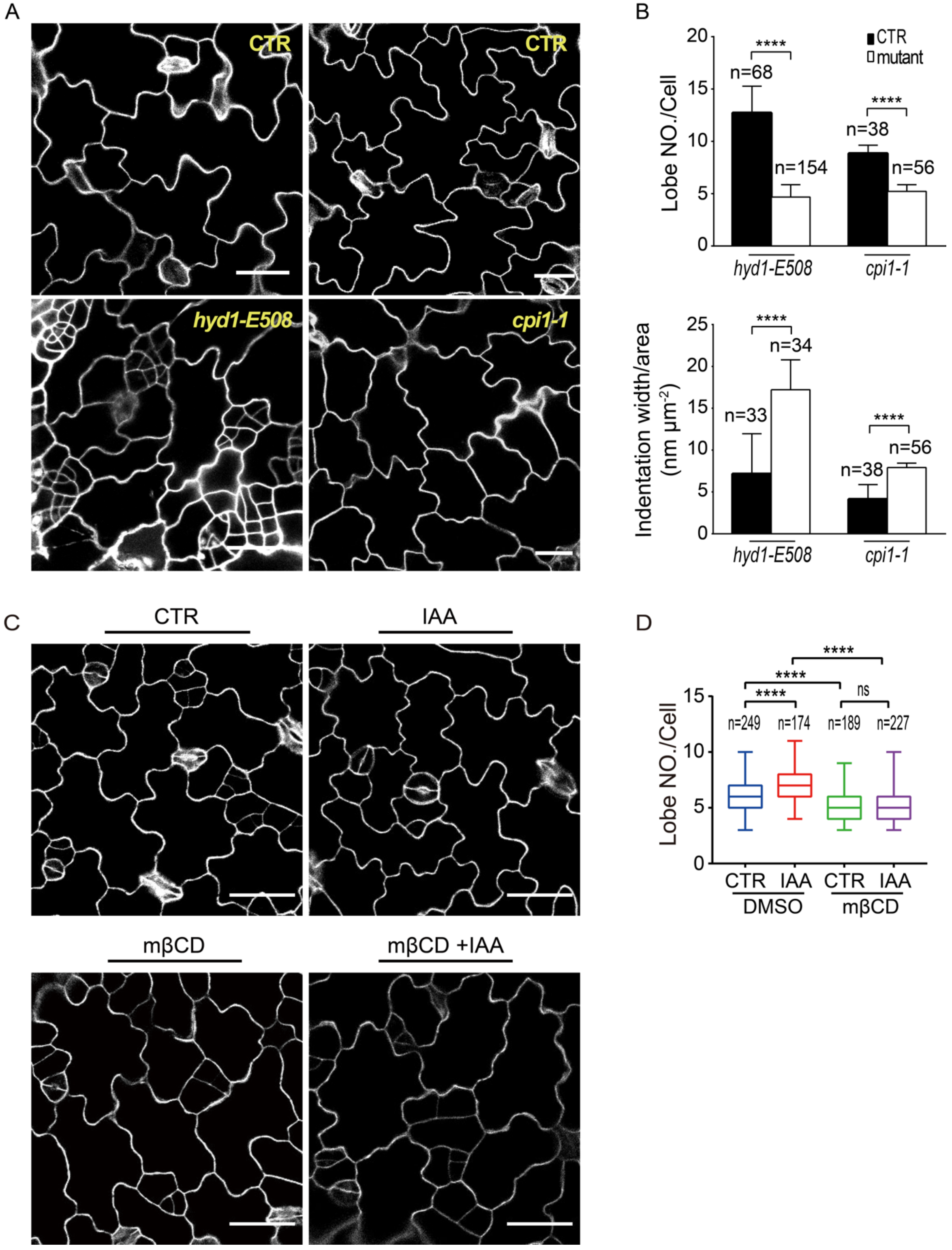
A
CTR
CTR
hyd1-E508
cpi1-1
B
Lobe NO./Cell
CTR
mutant
n=68
n=154
n=38
n=56
hyd1-E508
cpi1-1
Indentation width/area
(nm μm-2)
n=33
n=34
n=38
n=56
C
CTR
IAA
mβCD
mβCD +IAA
D
n=249
n=174
n=189
n=227
ns
CTR
IAA
CTR
IAA
DMSO
mβCD

Supplementary Figure 3. Sterol biosynthesis mutants show altered pavement cell (PC) shape. (A) Representative confocal images of cotyledon PCs in sterol biosynthesis mutants and their corresponding wild types. *HYDRA1* gene encodes a sterol Δ8-Δ7 sterol isomerase[1,2] and *FACKEL* gene encodes a sterol C14 reductase[3]. Cyclopropyl sterol isomerase1 (CPI1) functions to open the cyclopropyl ring of cycloeucalenol to produce obtusifoliol, the step in which the *cpi1-1* mutant is defective [4]. Homozygous *fk-J79*, *hyd1-E508* and *cpi1-1* mutants display near-sterile or sterile phenotype. Progenies derived from heterozygotes of each mutant are segregated into the wild-type (top panels) and homozygous mutant (bottom panels) phenotypes. PC phenotypes were analyzed at 7 days after seed plating. Scale bars = 30 µm. (B) Quantitative analyses of the number of lobes and indentation widths for sterol biosynthesis mutants and their wild types. The average lobe number per cell in mutants was fewer than their wild types, whereas the average indentation widths in mutants were significantly increased compared with that of their wild types. CTR, wild-type control. (C-D) Auxin-induced PC interdigitation was compromised in the presence of 10 mM methyl-β-cyclodextrin (mβCD). (C) Representative confocal images of PCs in wild-type (Col-0) cotyledons treated with DMSO (mock treatment), 50 nM IAA, 10 mM mβCD or 50 nM IAA plus 10 mM mβCD. CTR, control. Scale bars = 15 µm. (D) Quantitative analysis of the number of lobes under different treatments. Data in (B and D) are representative of three independent experiments with the same pattern and are presented as mean ± SD. *n* represents the number of independent cells.

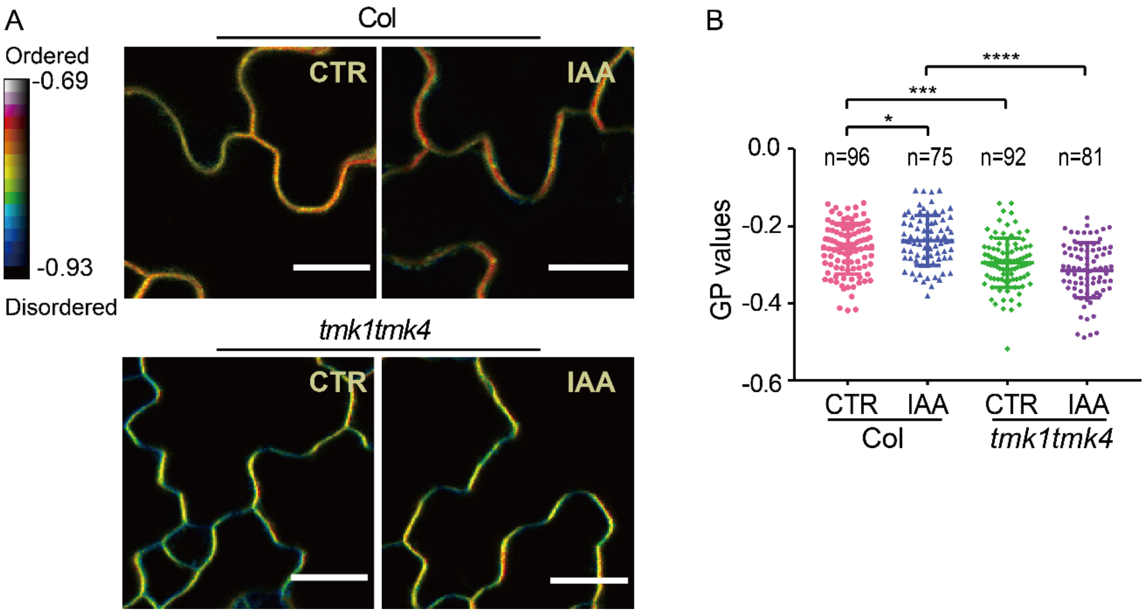

Supplementary Figure 4. The reduced membrane ordering in *tmk1tmk4* double mutant was not rescued by the exogenously supplied IAA. (A) Representative GP images of pavement cells in the wild type (*Col-0*) and *tmk1tmk4* double mutant with or without IAA treatments obtained after di-4-ANEPPDHQ staining. (B) Quantitative analysis of mean GP values extracted from the PM of multiple pavement cells from two independent experiments. Scale bars = 15 µm. *n* represents the number of independent cells. Data are presented as mean ± SD. CTR, control.

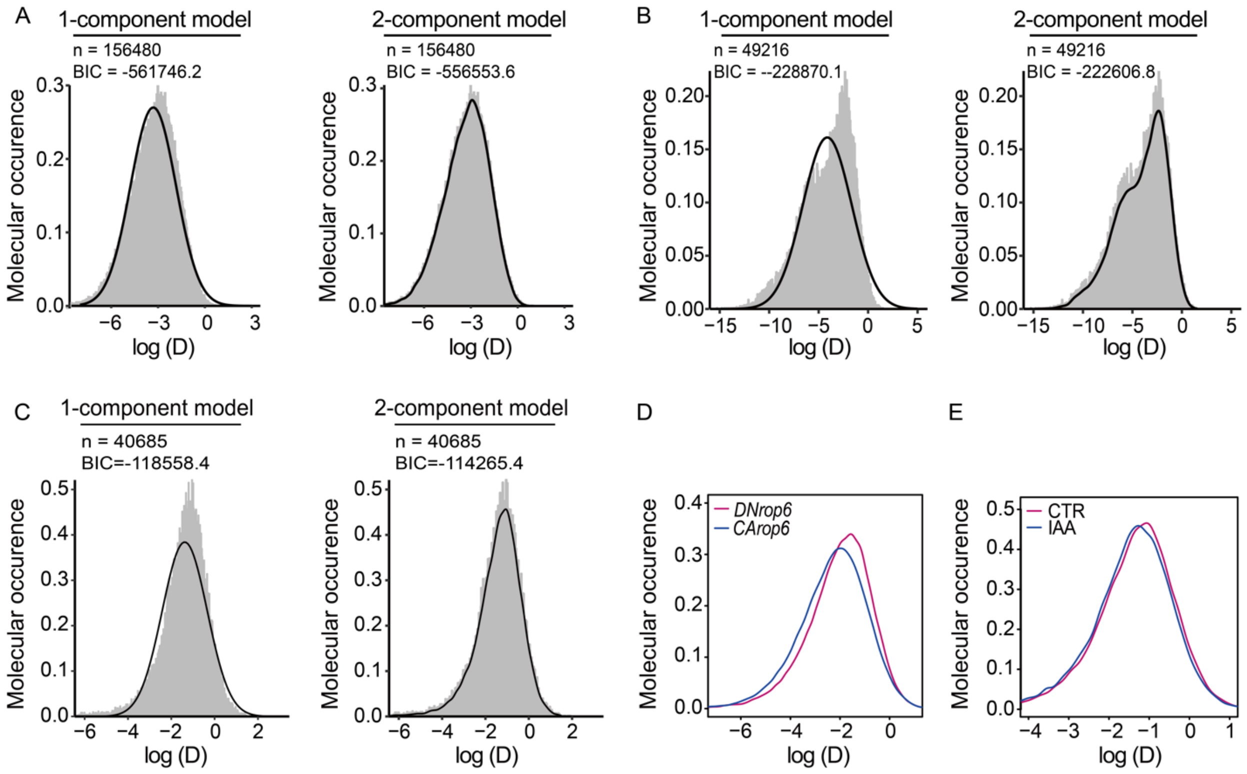

Supplementary Figure 5. Density histograms of diffusion coefficients of TMK1, flotillin1 and ROP6 particles. For all density distributions of TMK1 (A), flotillin1(B) and ROP6 (C) particles, the fitting of the density curve (solid line) to the density histogram (gray area) was significantly improved when the two-component mixture model was used. This is consistent with the higher Bayesian Information Criterion (BIC) values obtained from the two-component mixture model. The gray area indicates the density histogram of diffusion coefficients obtained by a mean squared displacement analysis of single-particle trajectories. The solid line shows the nonparametric density estimate generated by the density function in the R package mclust with the default settings. *n* represents the total number of particles. (D) The density curves of diffusion coefficients for YFP-*CArop6* (blue curve) and YFP-*DNrop6* (pink curve) particles. (E) The density curves of diffusion coefficients for mEGFP-ROP6 particles in cells with (blue curve) or without IAA treatment (pink curve). CTR, control.

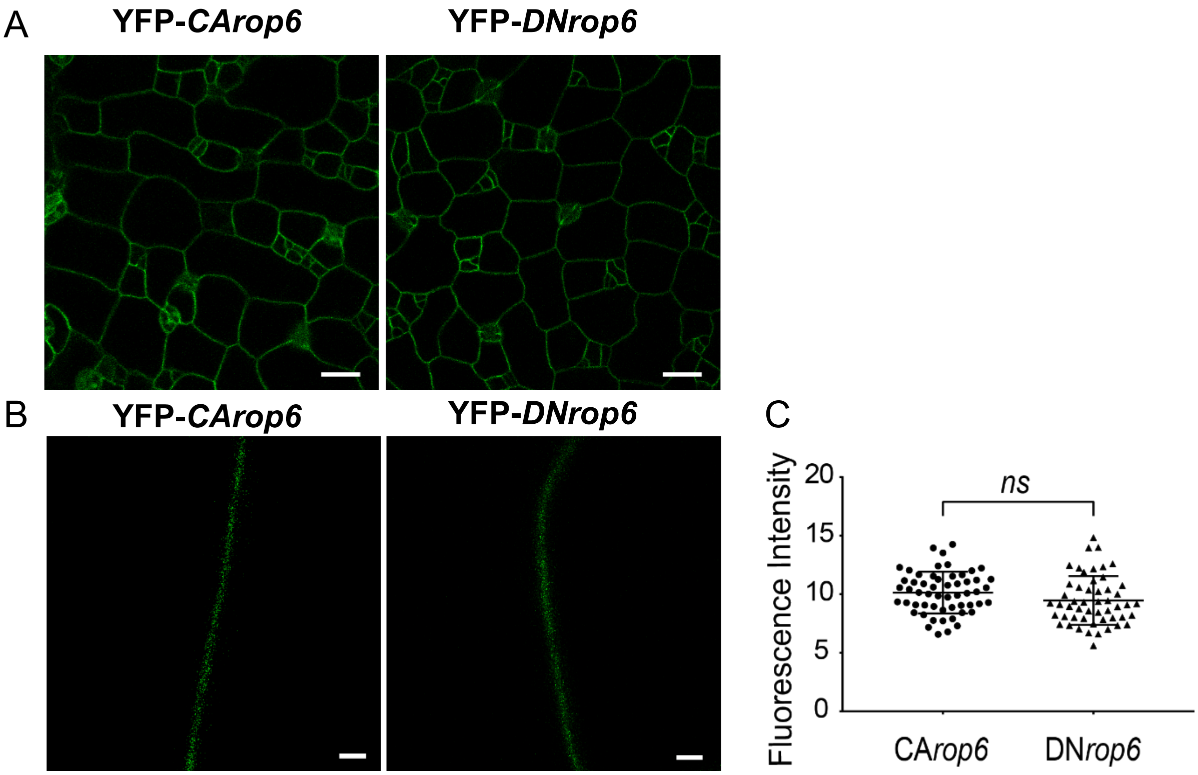

Supplementary Figure 6. Subcellular localization of 35S::YFP-*CArop6*^{Q64L} and 35S::YFP-*DNrop6*^{D121A} and the quantitative analysis. (A) Representative confocal images of pavement cells (PCs) of transgenic lines expressing 35S::YFP-*CArop6*^{Q64L} and 35S::YFP-*DNrop6*^{D121A} . (B) Magnified images of PCs expressing 35S::YFP-*CArop6*^{Q64L} and 35S::YFP-*DNrop6*^{D121A}. (C) Quantitative analysis showed that YFP-*CArop6*^{Q64L} and YFP-*DNrop6*^{D121A} have similar fluorescence intensity at the PM. Data were obtained from two independent experiments. Scale bars: 30 μm (A) and 2 μm (B)

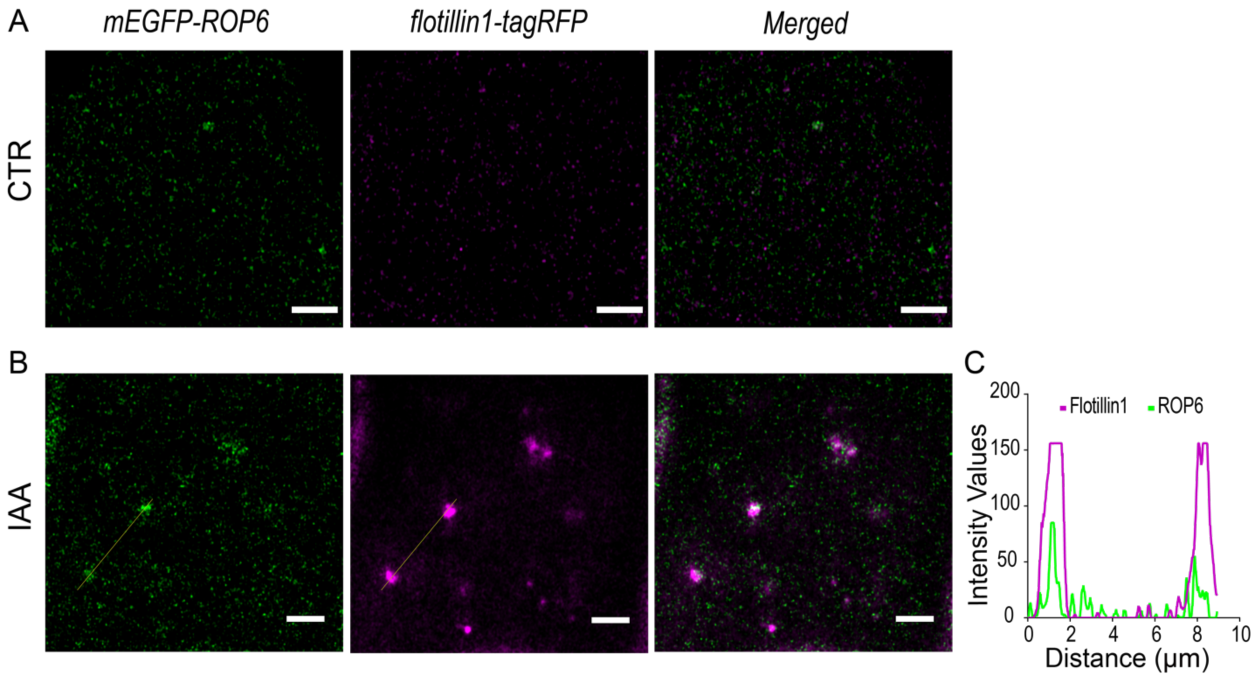

Supplementary Figure 7. ROP6 nanoclusters are partially colocalized with flotillin1 nanoclusters upon the IAA treatment. (A, B) Representative Airyscan images of pavement cells co-expressing ROP6::mEGFP-ROP6 and flotillin1::flotillin1-tagRFP without (A) or with IAA (B) treatments. (C) Line scan of fluorescence intensities as indicated in (B). When flotillin1-tagRFP was expressed under the flotillin1 native promoter, fluorescence was too weak to be detected in the control treatment. Therefore, we treated the transgenic cotyledons with IAA (1 μM, 10 mins), which promoted the clustering of flotillin1 particles and thus increased the fluorescence intensity of flotillin1-tagRFP. CTR, control. Scale bars: 3 μm.

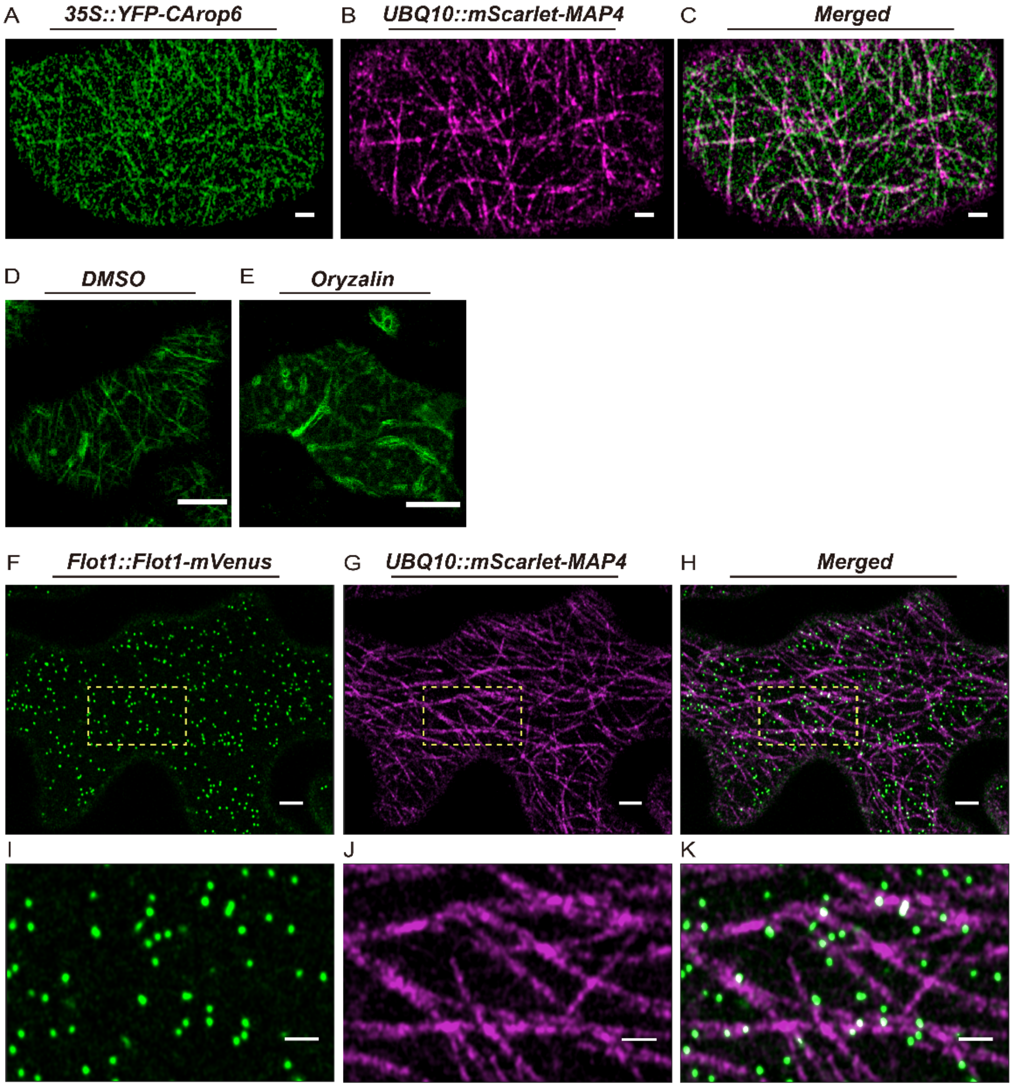

Supplementary Figure 8. CA*rop6* particles partially colocalize with cortical microtubules (CMTs) while the majority of flotillin1 particles are localized close to or within CMTs. (A-C) Representative confocal images showing the partial colocalization of CA*rop6* particles and microtubule associated protein-4 (MAP4). (A) YFP-CA*rop6* fluorescence. (B) mScarlet-MAP4 fluorescence. (C) Merged image. (D-E) Representative confocal images of pavement cells expressing GFP-β-tubulin (GFP-TUB) with the mock treatment (0.1% DMSO, v/v, 30 mins) or with oryzalin treatment (5 μM, 30 mins). (F-H) Representative confocal images showing that the majority of flotillin1 particles do not colocalize with CMTs. (F) Flotillin1-mVenus fluorescence. (G) mScarlet-MAP4 fluorescence. (H) Merged image. (I-K) Higher magnification of the regions indicated in the boxed areas in (F-H). White color reflects the overlap of mScarlet-MAP4 (magenta) with YFP-CA*rop6* (green) or flotillin1-mVenus (green). Scale bars: 2 μm (A-C), 15 μm (D-E), 3 μm (F-H) and 1 μm (I-K).

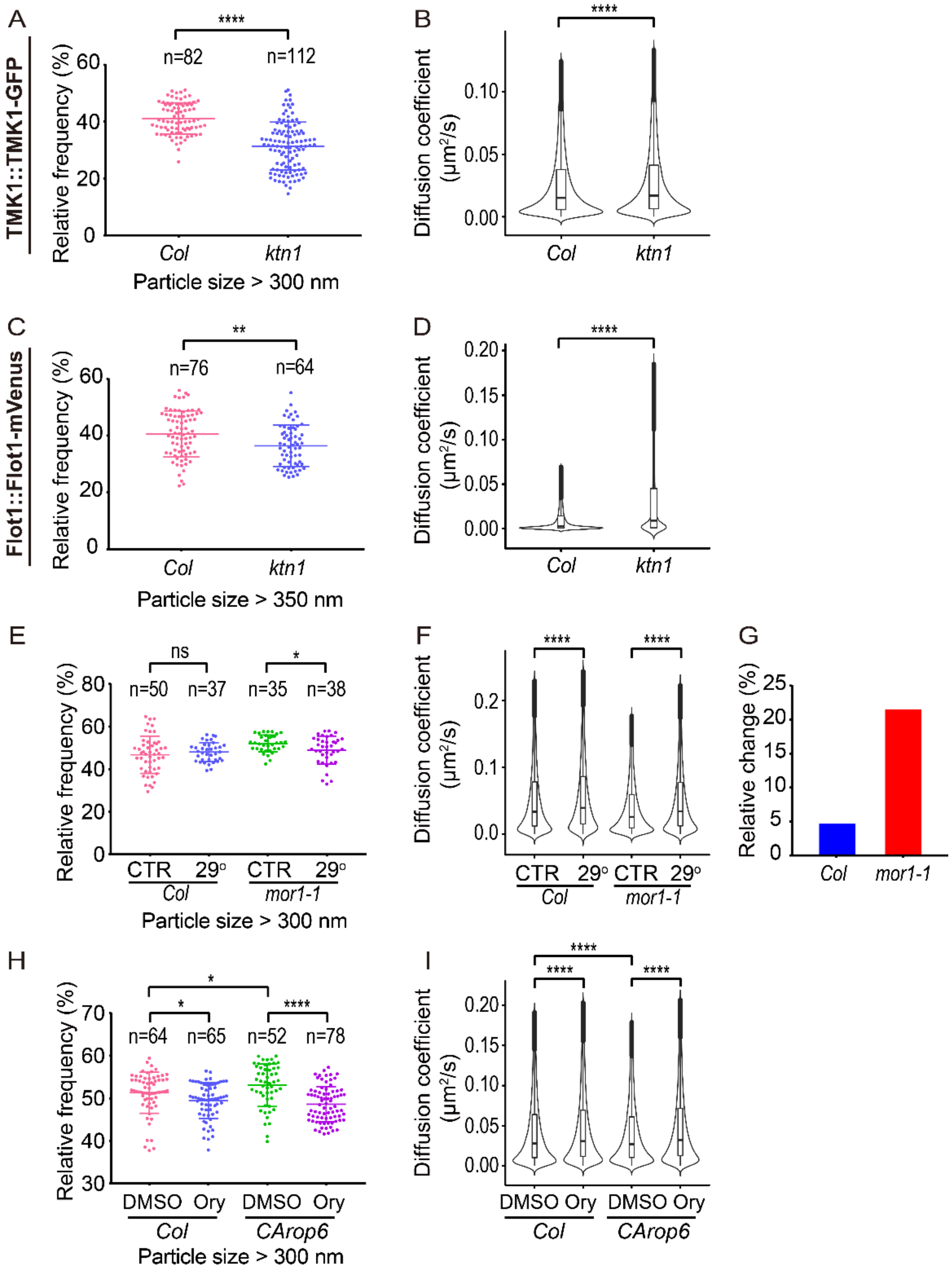
A
TMK1::TMK1-GFP
Relative frequency (%)
****
n=82
n=112
Col
ktn1
Particle size > 300 nm
B
Diffusion coefficient (μm²/s)
****
Col
ktn1
C
Flot1::Flot1-mVenus
Relative frequency (%)
**
n=76
n=64
Col
ktn1
Particle size > 350 nm
D
Diffusion coefficient (μm²/s)
****
Col
ktn1
E
Relative frequency (%)
ns
*
n=50
n=37
n=35
n=38
CTR
29°
CTR
29°
Col
mor1-1
Particle size > 300 nm
F
Diffusion coefficient (μm²/s)
****
****
CTR
29°
CTR
29°
Col
mor1-1
G
Relative change (%)
Col
mor1-1
H
Relative frequency (%)
*
*
****
n=64
n=65
n=52
n=78
DMSO
Ory
DMSO
Ory
Col
CArop6
Particle size > 300 nm
I
Diffusion coefficient (μm²/s)
****
****
****
DMSO
Ory
DMSO
Ory
Col
CArop6

Supplementary Figure 9. Microtubules regulate dynamic behaviors of TMK1 and flotillin1 particles at the plasma membrane. (A-D) Dynamics of TMK1-GFP and flotillin1-mVenus particles in wild-type *Col-0* and *ktn1* mutant devoid of KATANIN1 activity. (A) Relative frequency of TMK1 particles with size larger than 300 nm in *Col-0* and *ktn1* mutant. (B) Comparison of diffusion coefficients of TMK1 particles in *Col-0* and *ktn1* mutant. The number of particles analyzed in *Col-0* and *ktn1* mutant are106410 and 79628, respectively. (C) Relative frequency of flotillin1 particles with size larger than 350 nm in *Col-0* and *ktn1* mutant. (D) Comparison of diffusion coefficients of flotillin1 particles in *Col-0* and *ktn1* mutant. The number of particles analyzed in *Col-0* and *ktn1* mutant are 31030 and 27330, respectively. (E-G) Dynamics of TMK1-GFP particles in *Col-0* and *mor1-1* mutant at either 20 ^{o}C (permissive, control condition) or 29 ^{o}C (restrictive temperature). (E) Relative frequency of TMK1 particles with size larger than 300 nm. (F) Comparison of diffusion coefficients of TMK1 particles in different conditions. The number of particles analyzed in each treatment (from left to right) are 67735, 46983, 40253, and 47333, respectively. (G) The bar graph showing that the relative change of TMK1 diffusion coefficents is approximately 4-fold higher in *mor1-1* mutant than in *Col-0*. The relative change of diffusion coefficient is calculated by (diffusion coefficient at 29 ^{o}C - diffusion coefficient at 20 ^{o}C)/ diffusion coefficient at 20 ^{o}C. (H-I) Dynamics of TMK1-GFP particles in *Col-0* and CA*rop6* expression lines with or without oryzalin treatment. (H) Relative frequency of TMK1 particles with size larger than 300 nm. (I) Comparison of diffusion coefficients of TMK1 particles in different conditions. The number of particles analyzed in each treatment (from left to right) are 75768, 79288, 60135, and 95591, respectively. Ory, oryzalin. CTR, control. Data in A, C, E, H are presented as mean ± SD and *n* represents the number of independent cells. All data are representative of two independent experiments which have the same pattern.

Supplementary Table 1. Summary of diffusion characteristics of TMK1, flotillin1 and ROP6 particles, Related to Figures 3, 4, 5 and 6. Data are presented as mean ± SEM. Please refer to the Methods section for detailed calculations.

	Treatment	# of tracks	Population 1 (%)	Population 2 (%)	D_0 ($\mu m^2/s$)	D_1 ($\mu m^2/s$)	$D_{overall}$ ($\mu m^2/s$)
**Diffusion Characteristics of TMK1 particles, Related to Figure 3 and Figure 6**							
TMK1 in Col	DMSO	156480	51.39%	48.61%	0.050 ± 0.0005	0.131 ± 0.0006	0.089 ± 0.0004
TMK1 in Col	IAA	131187	50.72%	49.28%	0.042 ± 0.0004	0.122 ± 0.0007	0.082 ± 0.0004
TMK1 in Col	Oryzalin	85436	49.22%	50.78%	0.079 ± 0.001	0.162 ± 0.0009	0.121 ± 0.0007
TMK1 in Col	Oryzalin + IAA	96457	53.46%	46.54%	0.054 ± 0.0006	0.133 ± 0.0008	0.091 ± 0.0005
TMK1 in Col	mβCD	98267	48.85%	51.15%	0.083 ± 0.0009	0.179 ± 0.0009	0.1320± 0.0006
TMK1 in Col	mβCD + IAA	107269	47.22%	52.78%	0.077 ± 0.0008	0.181 ± 0.0008	0.1319± 0.0006
**Diffusion Characteristics of flotillin1 (Flot) particles, Related to Figure 4 and Figure 6**							
	Data presented in Figure 4						
Flot in Col	DMSO	49216	56.42	43.58	0.052±0.004	0.192±0.002	0.113±0.002
Flot in Col	IAA	43223	65.74	34.26	0.036±0.003	0.129±0.002	0.068±0.002
Flot in *tmk1tmk4*	DMSO	47078	34.73	65.27	0.374±0.039	0.831±0.006	0.672±0.012
Flot in *tmk1tmk4*	IAA	48206	33.93	66.07	0.396±0.052	0.848±0.007	0.695±0.014
	Data presented in Figure 6						
Flot in Col	DMSO	36937	51.28	48.72	0.065±0.006	0.154±0.003	0.109±0.003
Flot in Col	IAA	47316	52.52	47.48	0.025±0.002	0.118±0.002	0.069±0.002
Flot in Col	Oryzalin	53899	50.95	49.05	0.078±0.007	0.182±0.002	0.129±0.003
Flot in Col	Oryzalin + IAA	50214	52.59	47.41	0.055±0.005	0.140±0.002	0.095±0.003
**Diffusion Characteristics of ROP6 articles, Related to Figure 5**							
CArop6	DMSO	86712	35.30	64.70	0.162±0.003	0.260±0.002	0.225±0.002
DNrop6	DMSO	97390	28.93	71.07	0.166±0.003	0.299±0.001	0.261±0.001
ROP6 (wt) in Col	DMSO	40685	16.20	83.80	0.259±0.009	0.422±0.002	0.396±0.002
ROP6 (wt) in Col	IAA	42016	17.70	82.30	0.248±0.008	0.395±0.002	0.369±0.002
ROP6 (wt) in *tmk1tmk4*	DMSO	32663	15.32	84.68	0.372±0.013	0.636±0.004	0.596±0.004
ROP6 (wt) in *tmk1tmk4*	IAA	32893	15.68	84.32	0.378±0.011	0.652±0.004	0.609±0.004

Supplementary Table 2. Primers used for cloning and genotyping.

Primers name	Primer sequence
Primers used for cloning	
ROP6proF	GGGGACAAGTTTGTACAAAAAAGCAGGCTCCCAAGCTTTCAGAAAAGAGGATGATATAAG
ROP6proR	CTCGCCCTTGCTCACCATTCTAGACTTTCTCTCCTTCTTCAAACTTCAAAAACC
ROP6gF	CATGGACGAGCTGTACAAGGGCGGAGGAGGATCCATGAGTGCTTCAAGGTTTATCAAGTG
ROP6gR	GGGGACCACTTTGTACAAGAAAGCTGGGTCTCAGAGTATAGAACAACCTTTCTGAGATTT
mEGFPF	GGTTTTTGAAGTTTGAAGAAGGAGAGAAAGTCTAGAATGGTGAGCAAGGGCGAG
mEGFPR	CACTTGATAAACCTTGAAGCACTCATGGATCCTCCTCCGCCCTTGTACAGCTCGTCCATG
CArop6F	GGGGACAAGTTTGTACAAAAAAGCAGGCTCCATGAGTGCTTCAAGGTTTATCAAGTGTGT
CArop6R	GGGGACCACTTTGTACAAGAAAGCTGGGTCTCAGAGTATAGAACAACCTTTCTGAGATTT
flotmF	TATAAAGCTTGAACTGGTTGTTGATGATGATGATG
flotmR	TATAGGATCCCTTTTGATTTAATTTGGACTTTTCGCC
flotgF	TATAGGATCCATGTTCAAAGTTGCAAGAGCGTCAC
flotgR	GCTCCTCGCCCTTGCTCACCATAGATCCTCCTCCGCCGCTGCGAGTCACTTGCTTCGG
mVenusF	CCGAAGCAAGTGACTCGCAGCGGCGGAGGAGGATCTATGGTGAGCAAGGGCGAGGAGC
mVenusR	TATAGGTACCTTACTTGTACAGCTCGTCCATGCCG
FCF	TATAGGATCCATGTTCAAAGTTGCAAGAGCGTCAC
FCfusR	TCCTCCTCGCCCTTGCTCACCATTCTAGAGGATCCGCTGCGAGTCACTTGCTTCGGTTCC
FCfusF	CCGAAGCAAGTGACTCGCAGCGGATCCTCTAGAATGGTGAGCAAGGGCGAGGAGGATAAC
FCR	TATAGGTACCTTACTTGTACAGCTCGTCCATGCCGCC
LITF	TATAGGTACCATGAGTACAGCCACTTTCGTAG
LITmVenusF	GGGCGGAGGAGGATCTATGGTGAGCAAGGGCG
LITmVenusR	CGCCCTTGCTCACCATAGATCCTCCTCCGCCC
mVenusR1	TATAGGTACCTTACTTGTACAGCTCGTCCATG
TMK1F	GGGGACAAGTTTGTACAAAAAAGCAGGCTCCAGTTGGCTACACTCTTGTTTCCTC
TMK1R	GGGGACCACTTTGTACAAGAAAGCTGGGTCTCGTCCATCTACTGAAGTGAATGAC
ScarletF	CTCGGTACCCGGGGATCCTCTAGAGATGGTGAGCAAGGGCGAGG
ScarletR	GCCGGGAGCTTCCACTACCCTTGTACAGCTCGTCC
MAP4F	GTACAAGGGTAGTGGAAGCTCCCGGCAAGAAGA

MAP4R	TCTCACTCAGTTATTAACCTCCTGCAGGAAAGTGG
NOSF	TGCAGGAGGTTAATAACTGAGTGAGAATTTCCC
NOSR	CGTTGTAAAACGACGGCCAGTGCCACCGATCTAGTAACATAGATGAC
Primers used for genotyping	
*tmk1-1*F	TGAGCTCTCACTTCCTGAGAA
*tmk1-1*R	TGATCCTAGAGTGAAGTCTTT
*tmk4-1*F	TGCGATTGCTCAAAGAGGTCAGA
*tmk4-1*R	ATACGCCGGTAAGTTCATTC
mor1-1F	GCAGTAGACGTTATGTTTCAAGC
mor1-1R	TCTTTCGAGTAGGCTTGGCAC

References

1 Souter, M. *et al.* hydra Mutants of Arabidopsis are defective in sterol profiles and auxin and ethylene signaling. *Plant Cell* **14**, 1017-1031 (2002).

2 Gilk, S. D. *et al.* Bacterial colonization of host cells in the absence of cholesterol. *PLoS Pathog.* **9**, e1003107, doi:10.1371/journal.ppat.1003107 (2013).

3 Jang, J. C. *et al.* A critical role of sterols in embryonic patterning and meristem programming revealed by the fackel mutants of Arabidopsis thaliana. *Genes Dev.* **14**, 1485-1497 (2000).

4 Men, S. *et al.* Sterol-dependent endocytosis mediates post-cytokinetic acquisition of PIN2 auxin efflux carrier polarity. *Nat. Cell Biol.* **10**, 237-244, doi:10.1038/ncb1686 (2008).
