# Supplementary material for: Auxin-induced signaling protein nanoclustering contributes to cell polarity formation
Source: Nat Commun. 2020 Aug 6;11:3914. doi: 10.1038/s41467-020-17602-w (PMC7410848; doi:10.1038/s41467-020-17602-w)
Supplement: Supplementary file 3 — Descriptions of Additional Supplementary Files [file 41467_2020_17602_MOESM3_ESM.pdf]

Descriptions of Additional Supplementary Files

Supplementary Movie 1

Description: 3D reconstruction of cotyledon pavement cells stained with filipin. Filipin-sterol complexes appear to be more abundant at lobe-indentation regions. The 3D image was reconstructed from 11 optical sections spaced at 0.5 µm increments. Scale bar, 2.5 µm.

Supplementary Movie 2

Description: Example trajectories of flotillin1-mVenus particles on a 2-3-day-old Arabidopsis cotyledon pavement cell. The movie was acquired at 10 frames per second (100 ms exposure time) on a 256 x 256-pixels region of interest for 200 frames using TIRF microscopy. The dynamic behavior of flotillin1-mVenus particles was tracked by dragontrail command of IMARIS Track, in which 10 time points of the track length were shown. Trajectories are shown with color coding for absolute time to visualize the progressive dynamics. Scale bar, 2 µm.

Supplementary Movie 3

Description: Dynamic assembly and disassembly of flotillin1-mVenus particles on Arabidopsis cotyledon pavement cells. The movie was acquired at 10 frames per second (100 ms exposure time) for 100 frames using TIRF microscopy. Left Video: Representative video used for size measurement and single-particle tracking. Middle Video: Dynamics size-measurement of flotillin1-mVenus particles. The size of flotillin1-mVenus particles was measured using a region growing method in IMARIS. Right Video: Trajectories of flotillin1-mVenus particles. The dynamic behavior of flotillin1-mVenus particles was tracked by dragontrail command of IMARIS Track, in which 10 time points of the track length were shown. Trajectories are shown with color coding for absolute time to visualize the progressive dynamics. Scale bar, 0.2 µm.

Supplementary Movie 4

Description: 3D representation of a pavement cell expressing flotillin1-mCherry driven by the (CaMV) 35S promoter. After Agrobacteriummediated floral-dip transformation, we obtained a stable transgenic line with chimeric expression of the flotillin1-mCherry transgene. The sporadic expression of flotillin1- mCherry allows us to visualize the distribution of flotilllin1 proteins at the plasma membrane of single cells. The 3D image was reconstructed from 43 optical sections spaced at 0.55 µm increments. Scale bar, 20 µm.

Supplementary Movie 5:

Description: Example trajectories of TMK1-GFP particles on a 2-3- day-old Arabidopsis cotyledon pavement cell. The movie was acquired at 10 frames per second (100 ms exposure time) on a 256 x 256-pixels region of interest for 200 frames using TIRF microscopy. The dynamic behavior of TMK1-GFP particles was tracked by dragontrail command of IMARIS Track, in which 10 time points of the track length were shown. Trajectories are shown with color coding for absolute time to visualize the progressive dynamics. Scale bar, 2 µm.

Supplementary Movie 6

Description: Example trajectories of mEGFP-ROP6 particles on a 2-3- day-old Arabidopsis cotyledon pavement cell. The movie was acquired at 10 frames per second (100 ms exposure time) on a 256 x 256-pixels region of interest for 200 frames using TIRF microscopy. The dynamic behavior of mEGFP-ROP6 particles was tracked by dragontrail command of IMARIS Track, in which 10 time points of the track length were shown. Trajectories are shown with color coding for absolute time to visualize the progressive dynamics. Scale bar, 2 µm
